# Investigation of Solvatomorphism and Its Photophysical Implications for Archetypal Trinuclear Au_3_(1-Methylimidazolate)_3_

**DOI:** 10.3390/molecules26154404

**Published:** 2021-07-22

**Authors:** Shengyang Guan, David C. Mayer, Christian Jandl, Sebastian J. Weishäupl, Angela Casini, Alexander Pöthig

**Affiliations:** 1 Catalysis Research Center & Department of Chemistry, Technische Universität München, Ernst-Otto-Fischer Str. 1, D-85748 Garching b. München, Germany; shengyang.guan@tum.de (S.G.); david.mayer@tum.de (D.C.M.); christian.jandl@tum.de (C.J.); sebastian.weishaeupl@tum.de (S.J.W.); 2 Department of Chemistry, Technische Universität München, Lichtenbergstr. 4, D-85748 Garching b. München, Germany; angela.casini@tum.de

**Keywords:** cyclic trinuclear complex, photoluminescence, aurophilic interactions

## Abstract

A new solvatomorph of [Au_3_(1-Methylimidazolate)_3_] (Au_3_(MeIm)_3_)—the simplest congener of imidazolate-based Au(I) cyclic trinuclear complexes (CTCs)—has been identified and structurally characterized. Single-crystal X-ray diffraction revealed a dichloromethane solvate exhibiting remarkably short intermolecular Au⋯Au distances (3.2190(7) Å). This goes along with a dimer formation in the solid state, which is not observed in a previously reported solvent-free crystal structure. Hirshfeld analysis, in combination with density functional theory (DFT) calculations, indicates that the dimerization is generally driven by attractive aurophilic interactions, which are commonly associated with the luminescence properties of CTCs. Since Au_3_(MeIm)_3_ has previously been reported to be emissive in the solid-state, we conducted a thorough photophysical study combined with phase analysis by means of powder X-ray diffraction (PXRD), to correctly attribute the photophysically active phase of the bulk material. Interestingly, all investigated powder samples accessed via different preparation methods can be assigned to the pristine solvent-free crystal structure, showing no aurophilic interactions. Finally, the observed strong thermochromism of the solid-state material was investigated by means of variable-temperature PXRD, ruling out a significant phase transition being responsible for the drastic change of the emission properties (hypsochromic shift from 710 nm to 510 nm) when lowering the temperature down to 77 K.

## 1. Introduction

**Figure 1:**
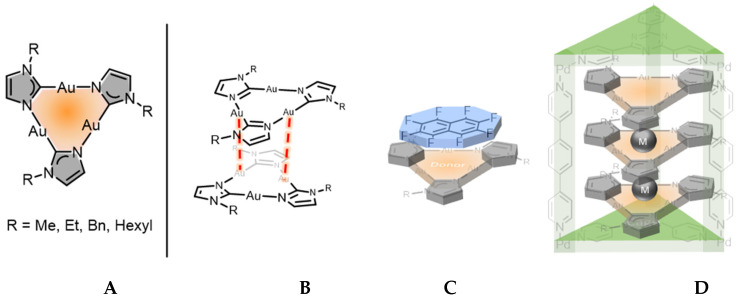
(**A**) General structure of imidazolate-based CTCs [Au_3_(MeIm)]_3_ [7,20]; [Au_3_(EtIm)]_3_ [3]; Au_3_(BzIm)_3_ [19]; Au_3_(HexIm)_3_ [18]; (**B**) illustration of possible metallophilic interaction (red dashed lines); (**C**) an example of π electron lewis acid/base binding model [18]; (**D**) guest supramolecular trigonal prismatic arrays within a Pd(II) coordination cage host, M = Ag^+^ cation [14].

Cyclic trinuclear complexes (CTCs) featuring three coordinated metal atoms in the nine-membered planar or near planar rings, represent some of the simplest polynuclear metal clusters. Comprehensive synthetic, structural, theoretical, and photophysical studies have been performed on a variety of cyclic trinuclear complexes, featuring series of angular ditopic bridging ligands, including carbeniate, pyrazolate, imidazolate, and triazolate ligands [1,2,3,4,5,6,7]. CTCs have found various applications in the field of intermolecular M(I)–M(I) interactions [8,9], metalloaromaticity/π-acidity and basicity [10], luminescence [11,12,13] and supramolecular assembly [14,15,16]. (Figure 1B–D) Imidazolate-based CTCs are the least studied compared to those of carbeniate and pyrazolate, only four examples have been reported (Figure 1A). In the 90s, Burini, Wagner and coworkers reported the synthesis of Au_3_ imidazolate CTCs bearing methyl or benzyl substituents, while more recently Omary et al. achieved complexes of general formula tris[(*μ*_2_-1-ethylimidazolato-N^3^,C^2^)Au(I)] (Au_3_(EtIm)_3_) (Et = ethyl) [3,7,17]. These complexes exhibit rich supramolecular chemistry having, e.g., fascinating luminescence properties in sandwich adducts of Au(I) imidazolate CTCs with Ag^+^ and Tl^+^ [6]. Very recently, CTCs were also shown to act as donors with a range of planar π-acceptors and exhibited exceptionally strong binding, which envisaged more complicated aromatic donor-acceptor supramolecular assembly(Figure 1C) [18,19]. Remarkably, Fujita et al. used the tris[(*μ_2_*-1-methylimidazolato-N^3^,C^2^)Au(I)] (abbreviated ‘Au_3_(MeIm)_3_′ in the following, Me = methyl) and Ag^+^ ions to form an unprecedented triple-decker ion cluster (Au_3_–Ag–Au_3_–Ag–Au_3_) within a self-assembled cage (Figure 1D) [14,15,16].

In this context, having great interest in small organometallic clusters [21,22], closed-shell coinage-metal-based systems with fascinating photo-physical properties [23,24], we further consider the Au(I) imidazolate-type CTCs as a valuable platform to investigate the luminescence, inter- and intra-trimer metal-metal interactions and related supramolecular assembly. The simplest scaffold - Au_3_(MeIm)_3_ was the first synthesized Au(I) imidazolate CTC by Burini in 1989 [7], but was not structurally determined until very recently by Ruiz et al. in 2020 [20]. The reported crystal structure showed no co-crystallization of solvent molecules or any pronounced intermolecular aurophilic interactions between Au_3_(MeIm)_3_. When reinvestigating the synthesis, we serendipitously discovered a new solvatomorph at an unconventional cryogenic crystallization condition, which actually features a dimer formation of Au_3_(MeIm)_3_ through aurophilic interactions. Such weak metallophilic interactions in linear-coordinate Au(I) complexes are similar to the energies of hydrogen bonds [8,25], and are also involved in the occurrence of polymorphism/solvatomorphism in the solid-state [26,27,28]. In addition, aurophilic interactions are closely related to luminescence [29], pointing towards significant differences in the emission properties of the different solid-state structures. Therefore, we conducted a combined photophysical and structural study, accompanied by DFT-calculations, of Au_3_(MeIm)_3_ being the archetypal molecule for imidazolate-based CTCs, to investigate which exact polymorph is the photophysically active phase.

## 2. Results and Discussion

We prepared Au_3_(MeIm)_3_ mainly following the route described previously by Burini [7] and Vaughan [30] with minor modifications (see Experimental section for details). Numerous attempts have been made to obtain crystals suitable for single-crystal X-ray diffraction analysis, however, the majority of attempts were unsuccessful. For example, slow evaporation of a solution of Au_3_(MeIm)_3_ in dichloromethane or chloroform solution at ambient conditions leads to the formation of a bright yellow solution and colloidal gold precipitation. To prevent decomposition, crystallization was then carried out at ultra-low temperature instead of reported room temperature crystallization. After keeping a saturated solution of dichloromethane/hexane (20:1) at 193 K (−80 °C) for two weeks, colorless needle-like crystals suitable for a single-crystal X-ray diffraction (SC-XRD) study were obtained.

The SC-XRD result reveals the presence of a novel solvatomorph (hereafter referred to as the solvatomorph **β**) which is structurally different from the one previously reported (the structure discovered by Ruiz et al. is referred as the polymorph **α**) [20]. In detail, in solvatomorph **β**, the trinuclear complex Au_3_(MeIm)_3_ crystallizes in triclinic system with space group *P*-1, featuring one co-crystallized solvent molecule of dichloromethane. The molecular structure of Au_3_(MeIm)_3_ can be considered identical for solvatomorphs **α** and **β**, within the means of statistical errors. The three gold atoms are bridged by the three 1-methylimidazolates with each metal ion being bound to one nitrogen and one carbon donor atom from a neighboring imidazolate. The angles of N-Au-C slightly deviate from linearity, which is in agreement to previously reported Au_3_ imidazolium trimers featuring bulkier substituents (ethyl/benzyl) [3,17]. The distances of intramolecular contacts are slightly exceeding the range of reported Au_3_ imidazolium complexes (ethyl/benzyl) (3.436(2) to 3.465(3) Å) [3,17].

**Figure 2:**
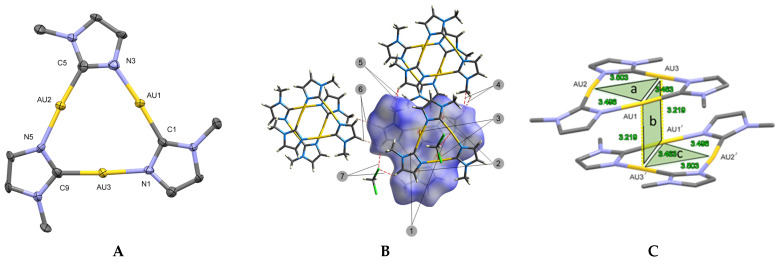
(**A**) A view of the molecular structure of the monomer of Au_3_(MeIm)_3_ from solvatomorph **β** with displacement ellipsoids shown at 50% probability. Hydrogen atoms are omitted for clarity. (note that the monomer triangle molecules are identical in both polymorphs. See Figure S9). (**B**) Overview of the different non-covalent interactions around Au_3_(MeIm)_3_ molecule within dimeric structure in solvatomorph **β** as determined by Hirshfeld surface analysis. (Note: Due to reasons of clarity not all symmetry equivalent interactions are depicted in the figure). (**C**) Dimer-of-trimer formation found in solvatomorph **β**. Solvent molecules are omitted for clarity. Selected bond lengths (Å): Au1⋯Au2, 3.4961(9) Å; Au2⋯Au3, 3.5032(8) Å; Au3⋯Au1, 3.4636(7) Å; Selected angles (Å) C-Au1-N, 175.8(4)°; C-Au2-N, 173.5(4)°; C-Au3-N, 175.3(1)°.

Despite the similarities in the molecular structure, the packing of Au_3_(MeIm)_3_ in solvatomorph **β** is fundamentally different. The Au_3_(MeIm)_3_ molecules show close contacts to one adjacent molecule, which can be attributed to attractive aurophilic interactions being absent in previously reported polymorph **α**. As a result, two Au_3_(MeIm)_3_ molecules are arranged in a twist-staggered fashion rotated by 180° relative to each other. To further understand the attractive interactions between the trimeric molecules, Hirshfeld surface analysis [31] was applied for one Au_3_(MeIm)_3_ molecule as shown in Figure 2B. In the d_normal_ mapping, four pairwise equivalent interactions (2 & 3) on the Hirshfeld surface between two trimers can be observed, which we attribute to two aurophilic interactions (3) and two short contacts (2) between carbon atoms on the heterocyclic rings. The interactions 1 and 7 are related to solvent molecules. Hydrogen bonds 7 (C-Cl⋯H) are formed between imidazolate back-bone hydrogen atoms and chlorine atoms of dichloromethane. Interaction 1 (C-H⋯Au distance: 2.7395 Å) resembles hydrogen bonds between dichloromethane and gold atoms, with CH group on dichloromethane acting as donor and gold acting as hydrogen bond acceptor. A survey using Cambridge Structural Database (CSD) was performed, revealing that among all the reported Au_3_ cyclotrimers preserving Au⋯H hydrogen bonding, Au_3_(MeIm)_3_ has the shortest distance between hydrogen to the triangular centroid. The Au⋯H distance (2.7395 Å) is shorter than the sum of the van der Waals radii (2.8 Å) [32]. In an authoritative review of the hydrogen bonding to gold, Schmidbaur et al. summarized different types of Au⋯H-X bonding. In the discussion of Au_3_ or Au_4_ clusters, he concluded this kind of bonding is dominated by classical Lewis-adduct formation [32,33,34]. Interactions 4~6 are CH⋯C hydrogen bonds bridging the imidazolate back-bone hydrogen atoms to methylene groups of neighboured, symmetry equivalent dimers.

The related Au_3_(EtIm)_3_ exhibits a similar stair-like dimer-trimer arrangement in the solid-state as solvatomorph **β** (compare Figure 2C) [3]. To quantify this structural similarity, we defined three planes that go through the hexanuclear gold core in the dimer-trimer in Figure 2C. Plane *a* is defined by Au1-Au2-Au3 on the top layer, plane *b* is defined by Au1-Au3 on the top and Au1-Au3 on the bottom, the plane *c* is defined by Au1-Au2-Au3 on the bottom. The plane angles ∠*ab* and ∠*bc* are both 88.07° in **β**, smaller than the plane angles in Au_3_(EtIm)_3_ (between 95.85 and 101.54°). The centroid *a* and centroid *c* are on parallel planes, their projections on the same plane are separated by 2.04 Å, while in the case of Au_3_(EtIm)_3_, the distance is 2.00 Å and 2.04 Å. The distance between plane *a* and *b* is shorter in Au_3_(EtIm)_3_ compared to solvatomorph **β** (3.01 Å vs. 3.18 Å), going along with shorter Au-Au distances and stronger aurophilic interactions (3.066(1) & 3.140(7) Å in Au_3_(EtIm)_3_).

**Figure 3:**
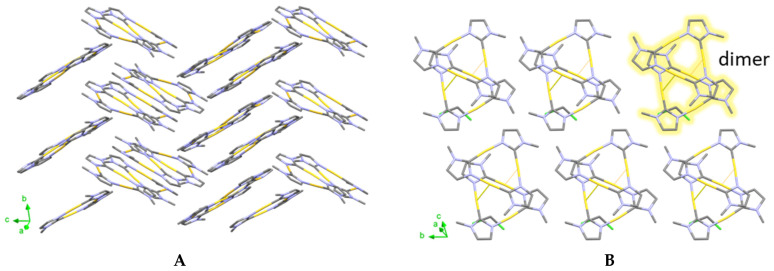
Packing of the different polymorphs of Au_3_(MeIm)_3_ in the solid-state: (**A**) Solvent-free polymorph **α**. (**B**) Solvatomorph **β** (dichloromethane solvate). Hydrogen atoms were omitted for clarity.

The differences between structure **α** and **β** in the intermolecular interactions consequently lead to the aforementioned different packing of the two crystal structures as shown in Figure 3. Au_3_(MeIm)_3_ molecules in the solvent-free monoclinic polymorph **α** are separated and form zigzag-like layers along the *c* axis (Figure 3A). Hereby, the intermolecular interactions are dominated by hydrogen bonding, and the closest Au-Au distance is 3.6660(5) Å [20]. In contrast, in the solvatomorph **β**, the dimers of Au_3_(EtIm)_3_ are packed within layered arrangements in the *b-c* plane (Figure 3B).

### 2.1. DFT Studies

The results of the SCXRD analysis of solvatomorph **β** together with the conducted Hirshfeld analysis clearly point towards the formation of intermolecular (additionally to coordination induced intramolecular) metallophilic Au-Au interactions in Au_3_(MeIm)_3_ trimer structures, which has never been observed so far. Therefore, to further rationalize the potential M-M interactions of the selected CTC, we performed electronic structure calculations by density functional theory (DFT), using the M06 meta-hybrid functional by Truhlar [35] and the CEP-31G(d) basis set [36]. This approach has successfully been applied in the theoretical description of CTCs [37,38] and other systems with predominantly non-covalent interactions [39]. Specifically, Galassi and co-workers used M06/CEP-31G(d) DFT computations to examine d^10^-d^10^ metal-metal bonding in the aforementioned CTC, [Au_2_(μ-C^2^,N^3^-MeIm)_2_Cu(µ-3,5-(CF_3_)_2_Pz)], which renders a direct comparison to related systems possible [37,40].

To study M-M interactions in solvatomorph **β**, the molecular structures of a monomeric and a dimeric CTC species were first optimized starting from the available crystallographic data. All optimized geometries were checked to be local minima of the potential energy surface (PES) by the absence of negative eigenfrequencies. The so obtained molecular structures are in good accordance with the crystallographic structures (Table 1). Lateral shifts of monomer in the computed dimeric structure are in excellent accordance with the monomer alignment found in the crystal structure.

Based on the optimized molecular geometries, we then accessed the nature of intra- and intermolecular aurophilic Au-Au interactions by following three methodologies: (i) calculation of the Gibbs free energy of the Au_3_(MeIm)_3_ dimer formation Δ*G_f_*, (ii) PES scan calculations to access the dimer dissociation energy *D_e_*, and finally (iii) bonding order (BO) analysis by calculations of different bond orders (among others Wiberg bond order = WBO [41], Mayer bond order = MBO [42], Fuzzy atom bond order = FBO [43] and assessment of bond critical points = BCP).

The HOMO of Au_3_(MeIm)_3_ is almost exclusively localized at the three imidazolate ligands with partial contribution from the gold atoms (p_π_-orbitals of the NHC ring and d_xy_ orbitals of the Au(I) atoms), whereas its LUMO shows strong contributions from the gold atoms with scarcely participation of the NHC ligands (Figure S1). The HOMO-LUMO gap accounts to 0.185 eV. For Au_3_(MeIm)_3_-{Dimer}, the HOMO shows a strong metal contribution, however with no pronounced electron density in intermolecular bonding regions. The LUMO of Au_3_(MeIm)_3_-{Dimer} reveals strong metal contributions, but with partial electron density located between two fragments, which suggests that intermolecular bonding might become stronger during photoexcitation (Figure S2). The dimer HOMO-LUMO gap accounts to 0.204 eV, as the HOMO level is lowered by 17.0 meV upon aggregation.

ESP analysis of Au_3_(MeIm)_3_-{Monomer} revealed a couple of minima and maxima at the electrostatic potential surface of the monomer species in ranges of −31.2 kcal/mol to 25.1 kcal/mol. The positions of these maxima are predominantly located at the hydrogen positions of the imidazolate backbone and the methyl substituent, with a global maximum found at the H11 position (Figure S3a). The global minima positions are mainly arising from abundant electron density below and above the positively charged Au_3_ triangle (Figure S3c). Assuming that only electrostatic interactions between monomers in Au_3_(MeIm)_3_-{Dimer} would govern the dimer formation, a T-shaped arrangement (enabling ideal contacts of regions of high and low ESP) would be expected. However, such behaviour is apparently not found in the dimer structure of **β**, which excludes strong electrostatic driven interactions between monomers of Au_3_(MeIm)_3_-{Dimer} being structurally decisive during crystallization, which is in agreement with the literature [10,38].

We further assessed Au-Au interactions in the dimer species by calculation of Δ*G_f_* and *D_e_*. The Gibbs free energy of formation was accessed via calculations of heats of formation following the reaction equation:

2 Au_3_(MeIm)_3_-{Monomer} → Au_3_(MeIm)_3_-{Dimer} (1)

By doing so we found a value for the dimer interaction energy Δ*G_f_*, = −20.48 kcal/mol, which is in the range expected for strong intermolecular aurophilic interactions (−10.24 kcal/mol per Au-Au bond) [8,44] and was recently found for comparable CTCs [37,38].

**Figure 4:**
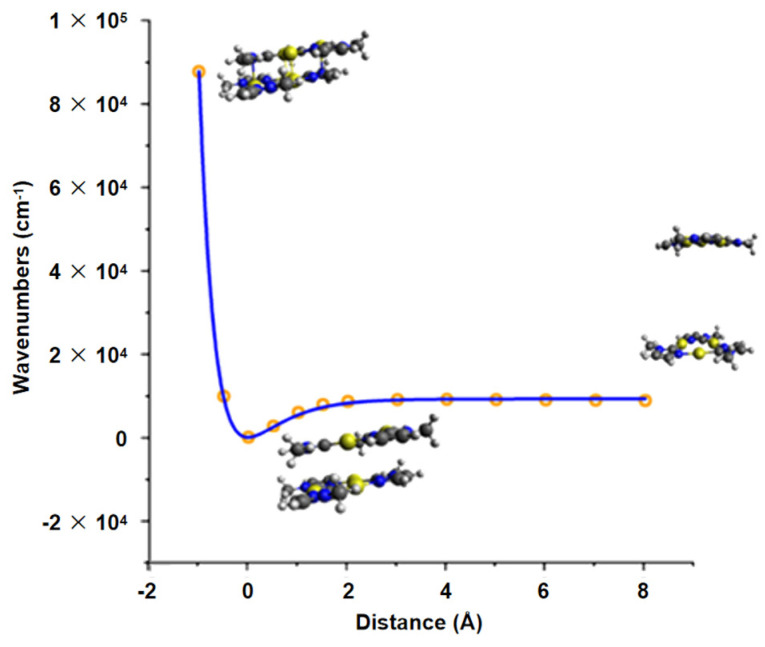
Results of the PES scan of the distance between two Au_3_(MeIm)_3_-{Monomer} in Au_3_(MeIm)_3_-{Dimer}. The inset shows three structures (−1 Å, 0 Å and 8 Å) at different distances. Note that we fitted a Morse potential to the single scan points for the extraction of *D_e_* (Orange circle: electronic energies from SP-DFT; blue line: fitting curve with function D_e_(1-exp(a(r-R_e_)))^2^ and R^2^ = 0.99976; the R_e_ value was set to 0 and the energies were corrected by the lowest energy structure).

To get access to the dimer dissociation energy *D_e_* PES scans along with the Au-Au distance in Au_3_(MeIm)_3_-{Dimer} (single point = SP calculations on vertically shifted Au_3_(MeIm)_3_-{Monomer} at similar level of theory, see Figure 4). Analysis of the so obtained PES revealed a *D_e_* of 9023 cm^−1^ or 26.3 kcal/mol, giving rise of 13.15 kcal/mol per intermolecular Au-Au bond. Such values are again expected for metallophilic Au-Au interactions and are consistent with the calculated Δ*G_f_*.

We finally calculated a number of different BOs, which are summarized in Table 2, distinguishing between Au-Au interactions within Au_3_(MeIm)_3_-{Monomer} and between Au_3_(MeIm)_3_-{Monomer} in the dimeric species (labeling according to crystallographic information). Based on purely crystallographic arguments, one would assume that aurophilic interactions between monomers are more pronounced, as bonding distances are smaller than those within a monomer complex (by polymorph **α** 0.3 Å). Hirshfeld analysis supports this argument, as the conducted BO analysis does. One clearly finds, that throughout all calculated indexes the BOs are doubled when going from intra- to intermolecular Au-Au interactions, evidencing a stronger intermolecular metallophilic binding in Au_3_(MeIm)_3_-{Dimer}. Consequently, the calculated BO indexes suggest appreciable metallophilic interactions to be responsible for dimer formation. (Note that the calculated BO values are in good agreement with literature values [8,45]).

In closing the theoretical section, all the above-presented results attest to the fact, that in Au_3_(MeIm)_3_-{Dimer} interactions between monomer species Au_3_(MeIm)_3_-{Monomer} are driven by robust aurophilic M-M interactions in accordance with the crystallographic analysis. Calculations of the dimer formation free energy underpinned by the dimer dissociation energy from PES scans, reveal rather strong aurophilic bonding interactions. Analysis of the bonding situation by different bonding indexes throughout reveals no negligible BOs, with a rather small bonding interaction within the Au_3_^3+^-core, but stronger interactions between monomer complexes in the dimer.

The aurophilic interactions are commonly associated with the luminescence properties of CTCs. Given that two different metal-metal bonding interactions can be found in the solid-state of Au_3_(MeIm)_3_, a thorough photophysical study in combination with phase analysis was conducted, to correctly correlate the photophysical active phase of the bulk material.

### 2.2. Qualitative Phase Analysis and Photophysical Studies

In the attempts to grow samples for single-crystal X-ray diffraction analysis, two crystallization methods were employed: (i) slow evaporation of dichloromethane solution of Au_3_(MeIm)_3_; (ii) vapor diffusion of *n*-hexane or diethyl ether into dichloromethane solution of Au_3_(MeIm)_3_. Additionally, crystallization experiments were carried out at different temperatures to prevent decomposition. Limited to the low diffusion coefficient of solvent at ultra-low temperature, vapor diffusion crystallization experiments with dichloromethane and hexane at 193 K did not yield suitable single crystals, while the slow evaporation of dichloromethane solution serendipitously offered a small number of single crystals of solvatomorph **β**. When applying temperates higher than 253 K (−20 °C) polymorph **α** is always yielded. Interestingly, dissolution of a solid sample of solvatomorph **β** in dichloromethane followed by evaporation higher than 253 K also leads to the formation of the **α** polymorph.

**Figure 5:**
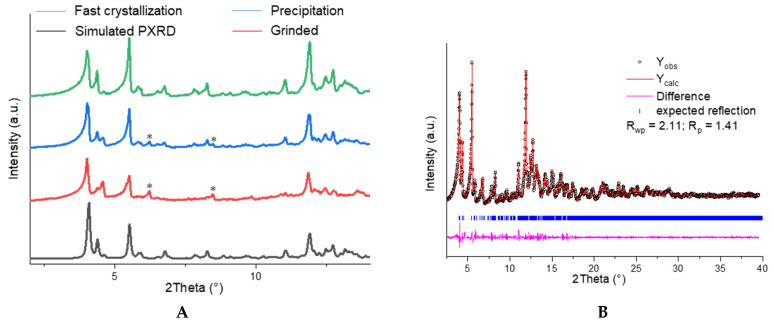
(**A**) Powder diffractograms (λ = 0.7093 Å) measured of samples obtained after fast crystallization (green), precipitation (blue), and grinded powder (red) at ambient conditions. A simulated pattern was generated from SC-XRD of polymorph **α** (black). (**B**) Pawley fit analysis of fast crystallization powder and calculated data. Observed data sets and calculated data are shown in black symbols and red line, respectively. The pink line shows the difference curve, while the blue marks show Bragg positions.

As the preparation of bulk crystalline samples for luminescence tests is generally conducted at ambient conditions, the formation of the **α** polymorph should be expected. However, since also different solvent combinations were used, compared to the preparations of the single crystals, we carefully investigated the solid-state phase of the prepared bulk samples by means of powder X-ray diffraction (PXRD). Hereby, products from (1) fast evaporation of Au_3_(MeIm)_3_ from a DCM/THF solution and (2) precipitation of Au_3_(MeIm)_3_ with *n*-hexane/Et_2_O from DCM/THF solution were characterized. The powder diffractograms of the samples of the two crystallization methods are shown in Figure 5A. Small differences can be visually observed (marked by asterisks), which we tentatively attribute to a minor side phase we cannot further specify. Pawley analysis was conducted on the measured powder data. The obtained cell parameters are in excellent agreement with the data from SC-XRD structures of polymorph **α** (Figure 5B). Consequently, all bulk crystallization methods at ambient conditions led to the formation of polymorph **α**, and fast crystallization by solvent removal was identified to yield excellent phase-pure material according to PXRD measurements, which consequently was used in the luminescence measurements described below.

The luminescence measurement of the crystalline sampe with polymorph **α** partially fits with previously reported results by Ruiz et al. When our sample is excited at 256 nm UV light, polymorph **α** exhibits an emission maximum at around 710 nm at room temperature. However, in their original work, Ruiz and co-workers report two emission bands, centered at 722 nm and 810 nm. With regard to our initial results in yielding phase-pure materials of polymorph **α** (compare above), our findings point toward the fact, that the 810 nm band in the originally reported samples might originate from contamination of a possible side phase, potentially exhibiting stronger aurophilic contacts. In all our experiments, we were not able to reproduce bulk materials showing the emission band at 810 nm. (Note that we were confirming phase-purity for all our samples by PXRD measurements before emission measurements, which was apparently not undertaken by Ruiz et al.) Finally, these results show, that a careful evaluation of bulk samples is of utter importance, when molecular properties are to be deduced from solid-state measurements.

**Figure 6:**
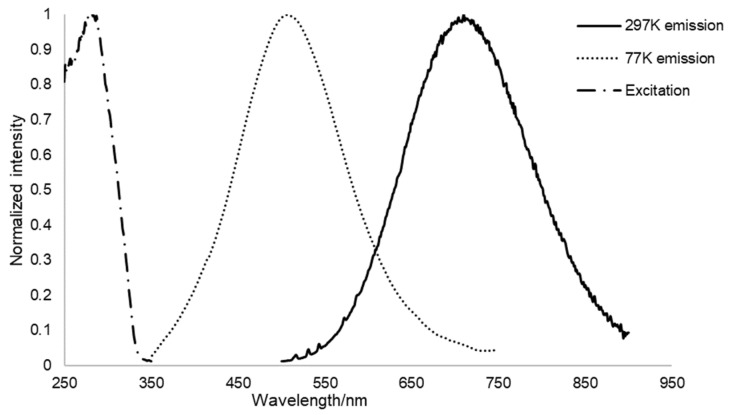
Diffuse reflectance and emission spectra of the solid samples of polymorph **α** at 297 K and 77 K. The excitation pattern is the same at room temperature and cryogenic temperature. Excitation peak centers at 256 nm, emission peak centers at 510 nm at 77 K, emission peak centers at 710 nm at 297 K. Quantum yield is 15% at 297 K and 60% at 77 K, respectively. The spectra have been normalized to 1 due to reasons of comparability.

**Figure 7:**
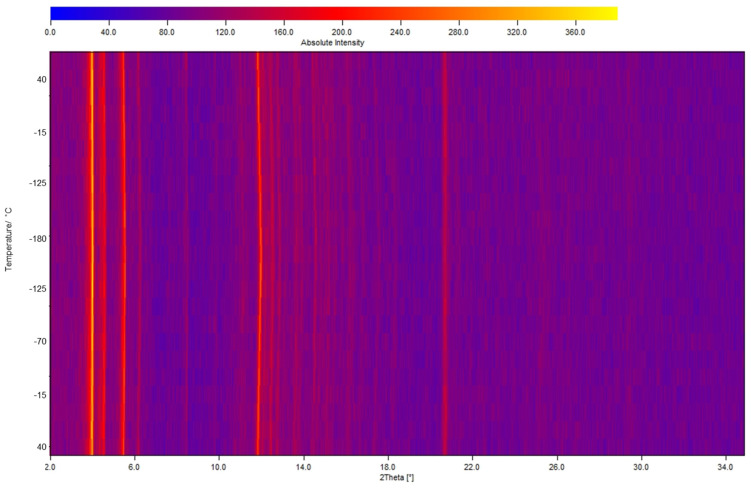
3D graphic illustration of the powder XRD VT series (λ = 0.7093 Å), the temperature goes from 40 °C to −180 °C and back to 40 °C.

During the course of the photophysical study, another attractive behavior of polymorph **α** was observed when cooling the sample with liquid nitrogen. At 77 K, the emission maximum moves to 510 nm with a dramatic intensity increase (quantum yield increased from 15% to 60%) (Figure 6). When warming up to 297 K, the original emission is restored and the whole process is fully reversible. Such a drastic hypsochromic shift at cryogenic temperature has already been observed for CTCs [3,46,47]. Raithby et al. considered a crystallographic phase transition as the possible origin in the temperature dependent luminescence shifts [48]. Therefore, we investigated possible structural changes or phase transitions with changing temperature. For the determination of the unit cell parameters at different temperatures, SC-XRD measurements were conducted first, showing that the crystal structure of polymorph **α** at 296(2) K remains in the same space group *P* 2_1_/*c* with <0.5% thermal expansion compared to when measured at 100(2) K. Accordingly, calculated crystal density decreases from 3.547 to 3.452 g/cm^3^, when the crystal is heated from 100 K to 296 K; intermolecular Au-Au distances slightly increase from 3.689 Å, 3.751 Å, 4.141 Å to 3.776 Å, 3.778 Å, 4.181 Å, respectively. All the intermolecular Au-Au distances significantly exceed the sum of the vdW radii of Au (3.32 Å) [8], no additional aurophilic interactions could be observed at cryogenic temperature. These findings are supported by non-ambient PXRD measurements (Figure 7), which also show no change in the diffraction pattern and therefore no phase transition when continuously measured between −180 °C and 40 °C. Also, the thermal expansion of the crystal structure does not show a discontinous change in the cell volume (See ESI Figure S12), therefore also not indicating a phase transition. Hence, we think that the minor structural changes in the packing of polymorph **α** cannot be correlated with the observed distinct thermochromism.

Another possible explanation for the thermochromism in CTCs have been hypothesized, e.g., by Omary and coworkers who observed similar thermochromism for Au_3_EtIm_3_ [3,37]. In detail, the related emission band centered at 425 nm at T < 200 K shifts to 700 nm at T ≥ 200 K. They suggested that at a higher temperature (above 200 K), a non-radiative relaxation to an intermediate excitation state takes place, which then gives rise to the low energy emission band. At lower temperatures (T < 200 K) emission from an energetically higher excitation state is supposed to happen, leading to the observed band centered at 425 nm. Since we did not see any significant structural changes in the solid-state at low temperatures, this might also serve as a viable explanation of our observations for Au_3_(MeIm)_3_.

## 3. Materials and Methods

All manipulations were carried out under an inert atmosphere of argon using standard Schlenk line. The preparation of Au_3_(MeIm)_3_ followed the route described previously by Burini [7] and Vaughan [30] with minor modifications: The 1-methylimidazole was freshly distilled and degassed before use. Hexane and THF were dried using a MBraun MBSPS 5 apparatus and stored over 4 Å molecular sieves. All other reagents were used as supplied. NMR spectra were recorded on a Bruker AV400 spectrometer at room temperature. ^1^H NMR spectra were referenced to the signals of CDCl_3_. 2 mg of 1-methylimidazole was dissolved in tetrahydrofuran, then cooled to −30 °C. To the stirred solution of methylimidazole in tetrahydrofuran, one equivalent of n-butyllithium in hexane was added drop-wisely under argon. The pale-yellow mixture was kept stirring for 1.5 h at −30 °C, before the addition of the equal equivalent amount of chloro(tetrahydrothiophene)gold(I) in 5 mL tetrahydrofuran. The reaction was kept at −30 °C for another hour, then 0.5 mL of dry methanol was added to quench the reaction. Afterward, the mixture was evaporated to dryness at 0 °C. The precise temperature control and anaerobic condition are obligatory for the deprotonation and metalation to happen. The remaining solid was dissolved in dichloromethane then filtered with Celite. Recrystallization in dichloromethane offered the analytical product. ^1^H NMR (400.13 MHz, CDCl_3_, 298 K): δ (ppm) = 7.15 (s, 3H, *H_NCHC_*), 6.96 (s, 3H, *H_NCHC_*), 3.81 (s, 9H, *H_CH3_*). ^13^C{^1^H} NMR (100.62 MHz, CDCl_3_, 298 K): δ (ppm) = 168.51, 127.67, 119.89, 35.97. ESI-MS (*m*/*z*): 834.60 [Au3(MeIm)3]^+^, 917.10 [Au3(MeIm)3]MeIm^+^_,_ 1471.09 [Au3(MeIm)3][Au_2_MeIm_3_]^+^. Elemental analysis: Calculated for C_12_H_15_Au_3_N_6_: C, 17.28; H, 1.81; Au, 70.83; N, 10.07. Found: C, 17.36; H, 1.87; N, 9.77. UV-Vis (solid-state, nm): 222, 259, 276.

## 4. Conclusions

In conclusion, this study revealed a new solvatomorph **β** (dichloromethane solvate) of Au_3_(MeIm)_3_, the archetypal imidazolate CTC molecule, exhibiting unprecedented short intermolecular aurophilic interactions in the solid-state which were rationalized with DFT calculations. Crystallization conditions were investigated in combination with powder X-ray diffraction monitoring to correctly correlate the photophysically active phase of bulk crystalline material. All investigated powder samples accessed via different preparation methods can be assigned to the pristine solvent-free polymorph **α**, showing no aurophilic interactions. In addition, a strong thermochromic behavior of polymorph **α** was observed, going along with no significant structural changes in the solid state at low temperatures. This indicates, that the observed thermochromism might be originating from a change in the effective emission pathways of the molecular Au_3_(MeIm)_3_ rather than emerging from a phase transition alongside with change in the intermolecular interactions. From a more generalized perspective, this study provides the first example of solvatomorphism induced aurophilic interactions in cyclic trinuclear complexes, and underlines the importance of a thorough solid-state characterization when deducing properties of molecular materials.

## Figures and Tables

**Table 1:** Comparison of calculated results from density functional theory (DFT) and experimental data from SC-XRD.

	Experimental Data	Calculated Results
Au-N bond/Å	2.030(9), 2.036(9), 2.043(8)	2.091
Au-C_carb_ bond/Å	1.986(11), 1.987(11), 1.988(11)	2.038
C_carb_-Au-N angle/°	173.6(4), 175.3(4), 175.8(4)	175.13
intratrimer Au1-Au2/Å	3.4961(9)	3.567
intratrimer Au1-Au3/Å	3.4636(7)	3.555
intratrimer Au2-Au3/Å	3.5032(8)	3.584

**Table 2:** Results of the BO analysis showing the results of different bonding indexes (WBO = Wiber bonding order, MBO = Mayer bonding order, FBO = Fuzzy bonding order, BCP = bond critical point).

	WBO	MBO	FBO	BCP
Au1-Au2 (intra)	0.135	0.062	0.190	no
Au1-Au3 (intra)	0.135	0.079	0.192	no
Au1-Au2-Au3 (intra)	---	0.035 (normalized multi-center BO)	---	yes
Au1-Au3 (inter)	0.269	0.156	0.428	yes

## Data Availability

CCDC 2093115-2093117 contain the supplementary crystallographic data for this paper. These data can be obtained free of charge from The Cambridge Crystallographic Data Centre via www.ccdc.cam.ac.uk/structures.

## Supplementary Materials

The following are available online. Details on: DFT calculations, single-crystal X-ray determinations, Hirshfeld surface analyses, and spectroscopic measurements.

## Sample Availability

Samples are not available from the authors.
