# Supplementary material for: Investigation of Solvatomorphism and Its Photophysical Implications for Archetypal Trinuclear Au3(1-Methylimidazolate)3
Source: Molecules. 2021 Jul 22;26(15):4404. doi: 10.3390/molecules26154404 (PMC8348911; doi:10.3390/molecules26154404)
Supplement: Supplementary file 1 [file molecules-26-04404-s001.zip › molecules-1301731-SI.pdf]

Supporting Information

Investigation of Solvatomorphism and Its Photophysical Implications for Archetypal Trinuclear Au_3(1-Methylimidazolate)$_3$

Shengyang Guan[1,2], David C. Mayer[1], Christian Jandl[1], Sebastian J. Weishäupl[1], Angela Casini[2] and Alexander Pöthig[1*]

1. Catalysis Research Center & Department of Chemistry, Chair of Inorganic and Metal-Organic Chemistry,
Technische Universität München, Ernst-Otto-Fischer Str. 1, D-85748 Garching b. München, Germany.

2. Department of Chemistry, Chair of Medicinal and Bioinorganic Chemistry, Technische Universität München, Lichtenbergstr. 4, D-85748 Garching b. München, Germany.

1. DFT analysis result 2
2. Hirshfeld surface analysis 2-D fingerprint plots 7
3. Crystallographic details 8
4. Spectral results 21
5. Solid-state emission spectra of polymorph α 23
6. Reference 24

1. DFT analysis result

All electronic structure calculations in this work were performed on the level of density functional theory (DFT). Ground state structures of the examined molecules were optimized using the GAUSSIAN 16 program at the M06/CEP31G(d) level of theory.[1-5] We used the crystallographic information to create input geometries. Note, that we added another d-polarization function to the main group elements. The structures were checked to be local minima of the PES by absence of negative eigenfrequencies by evaluation of the Hessian. The PES was scanned starting from the found ground state structure of the dimeric form subsequently artificially elongating the orthogonal distance between the two monomers at steps of 0.5 Å (for distances near the minimum) and 1.0 Å (for distances far from minimum). Energies were extracted with the help of single point calculations on the respective structures. We fitted a Morse potential to these points to extract the dimer dissociation energy. Analysis of the bonding orders, critical bonding points and the electrostatic surface potential was conducted using the Multiwfn software.[6]

1.1 Coordinates

$[Au_3(MeIm)]_3$-[1] (monomer)

Nimag = 0

0 1

Au	1.66079	1.21459	0.00015
Au	0.22176	-2.04504	-0.00002
Au	-1.88249	0.83079	-0.00006
N	-0.96951	2.70744	-0.00004
N	0.69295	4.18280	-0.00016
N	2.83005	-0.51425	0.00018
N	3.27575	-2.69179	-0.00010
N	-1.86003	-2.19347	-0.00015
N	-3.96902	-1.49152	0.00011
C	0.40474	2.81805	0.00006
C	-1.53698	3.99147	-0.00012
C	-0.50107	4.92482	-0.00014
C	2.04284	4.76117	-0.00030
C	2.23839	-1.75944	-0.00004
C	4.22583	-0.66529	0.00008
C	4.51565	-2.02930	-0.00007
C	3.10102	-4.14994	-0.00026
C	-2.64314	-1.05870	-0.00006
C	-2.68821	-3.32707	-0.00006
C	-4.01446	-2.89658	0.00007
C	-5.14509	-0.61201	0.00032
H	-2.61287	4.14389	-0.00012
H	-0.50408	6.01176	-0.00019
H	2.76796	3.93232	0.00003

H	2.19665	5.37902	0.89974
H	2.19677	5.37842	-0.90074
H	4.89612	0.18998	0.00021
H	5.45823	-2.57059	-0.00020
H	3.55939	-4.59216	-0.90016
H	2.02056	-4.36297	-0.00109
H	3.55799	-4.59227	0.90031
H	-2.28223	-4.33502	-0.00016
H	-4.95426	-3.44267	0.00013
H	-5.75694	-0.78773	-0.89981
H	-4.79024	0.43050	0.00024
H	-5.75661	-0.78771	0.90069

$[Au_3(MeIm)]_3$-[1] (dimer)

Nimag = 0

Au	0.21709	1.32871	-1.66654
Au	3.49010	0.68699	-0.35413
Au	1.29976	-2.01634	-1.13824
N	-0.53008	-1.57746	-2.04697
N	-2.17745	-0.30599	-2.82552
N	1.51372	2.86166	-1.07060
N	3.32326	3.79755	-0.18254
N	4.06169	-1.30818	-0.10408
N	3.78173	-3.50940	0.02982
C	-0.91964	-0.26778	-2.22734
C	-1.53865	-2.42933	-2.51931
H	-1.44603	-3.51203	-2.46545
C	-2.57812	-1.63997	-3.01054
H	-3.53722	-1.89442	-3.45553
C	-2.98432	0.86983	-3.17372
H	-2.42347	1.77021	-2.87371
H	-3.94112	0.84996	-2.62240
H	-3.17481	0.89984	-4.25962
C	2.76078	2.57768	-0.56212
C	1.28878	4.24414	-0.99906
H	0.36151	4.69070	-1.35006
C	2.41992	4.84288	-0.44249
H	2.66113	5.88051	-0.22560
C	4.63541	3.95818	0.45266
H	5.25465	4.67172	-0.11556
H	5.12941	2.97361	0.46474
H	4.51969	4.31660	1.49074
C	3.15387	-2.31882	-0.34156
C	5.24866	-1.86132	0.40429

H	6.11038	-1.24534	0.64919
C	5.08197	-3.24347	0.49443
H	5.74979	-4.03472	0.82564
C	3.17275	-4.84271	-0.05849
H	3.79640	-5.50912	-0.67678
H	2.18273	-4.73161	-0.52898
H	3.05212	-5.28086	0.94673
Au	-0.21695	-1.32873	1.66650
Au	-3.48997	-0.68706	0.35401
Au	-1.29985	2.01637	1.13817
N	0.53002	1.57755	2.04679
N	2.17747	0.30619	2.82532
N	-1.51354	-2.86181	1.07066
N	-3.32310	-3.79756	0.18248
N	-4.06163	1.30811	0.10407
N	-3.78226	3.50946	-0.02916
C	0.91966	0.26791	2.22716
C	1.53852	2.42950	2.51917
H	1.44582	3.51219	2.46530
C	2.57801	1.64021	3.01045
H	3.53710	1.89472	3.45543
C	2.98442	-0.86959	3.17348
H	2.42447	-1.76992	2.87168
H	3.94201	-0.84863	2.62357
H	3.17336	-0.90068	4.25962
C	-2.76054	-2.57772	0.56207
C	-1.28874	-4.24432	0.99920
H	-0.36161	-4.69102	1.35038
C	-2.41988	-4.84296	0.44252
H	-2.66121	-5.88058	0.22575
C	-4.63516	-3.95810	-0.45293
H	-5.25444	-4.67178	0.11507
H	-5.12919	-2.97354	-0.46486
H	-4.51931	-4.31630	-1.49107
C	-3.15402	2.31892	0.34171
C	-5.24879	1.86110	-0.40401
H	-6.11035	1.24496	-0.64907
C	-5.08250	3.24332	-0.49367
H	-5.75058	4.03450	-0.82454
C	-3.17394	4.84300	0.06009
H	-3.79657	5.50793	0.68100
H	-2.18278	4.73179	0.52814
H	-3.05595	5.28297	-0.94465

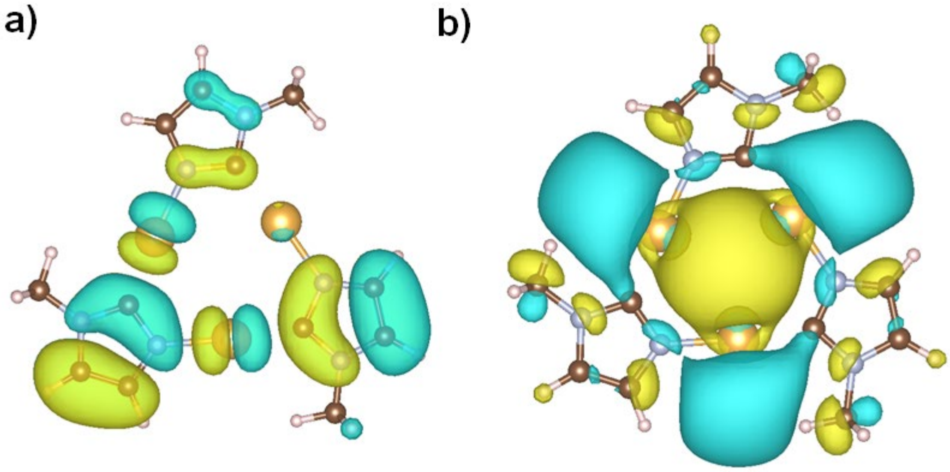

Figure S1. (a,b) KS-HOMO (left) and KS-LUMO (right) of $[Au_3(MeIm)]_3$-[1]. Isodensity is 0.02.

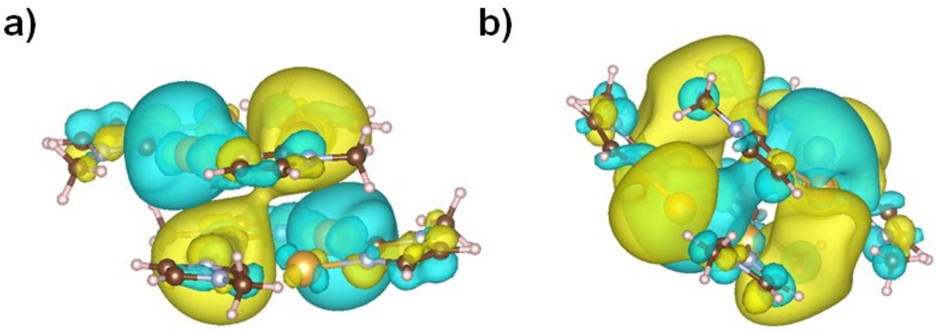

Figure S2. (a,b) KS-HOMO (left) and KS-LUMO (right) of $[Au_3(MeIm)]_3$-[1]. Isodensity is 0.02.

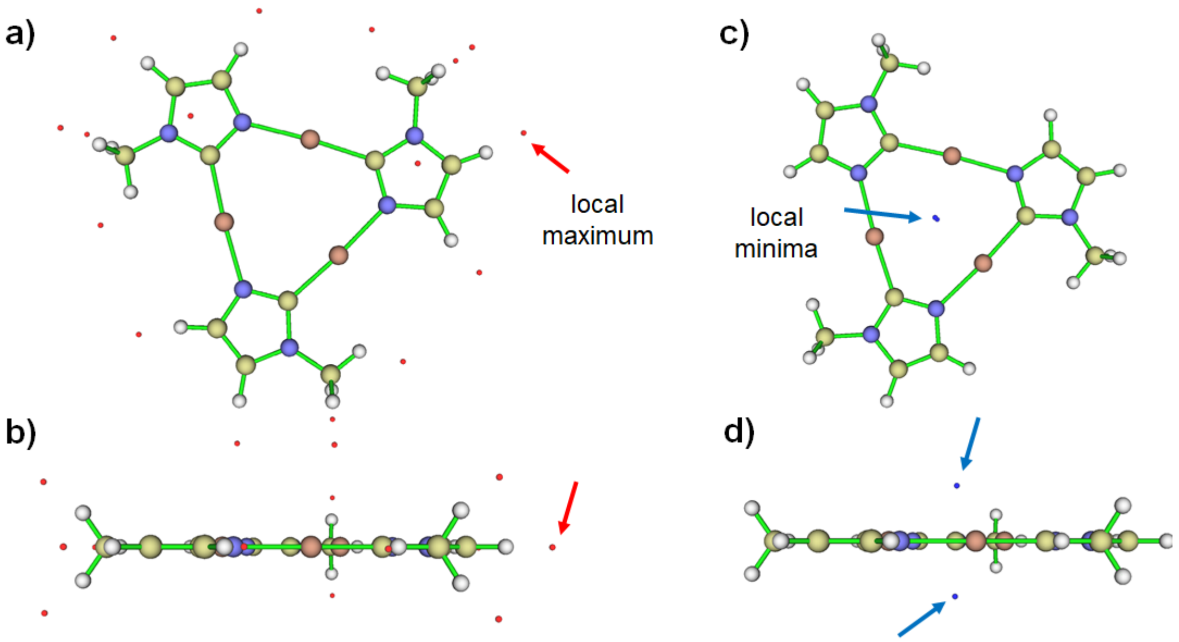

Figure S3. (a,b) Positions of the ESP maxima (red dots) and the corresponding local maximum (red arrow) from above and in side view showing electron density accumulation in the out region of the monomer. (c,d) Positions of the local ESP minima up and above of the monomer (blue dots and arrow). Note that pure electrostatic driven interaction would lead to an overlap of the local maxima and minima ESP positions and thus give rise of a T-shaped arrangement.

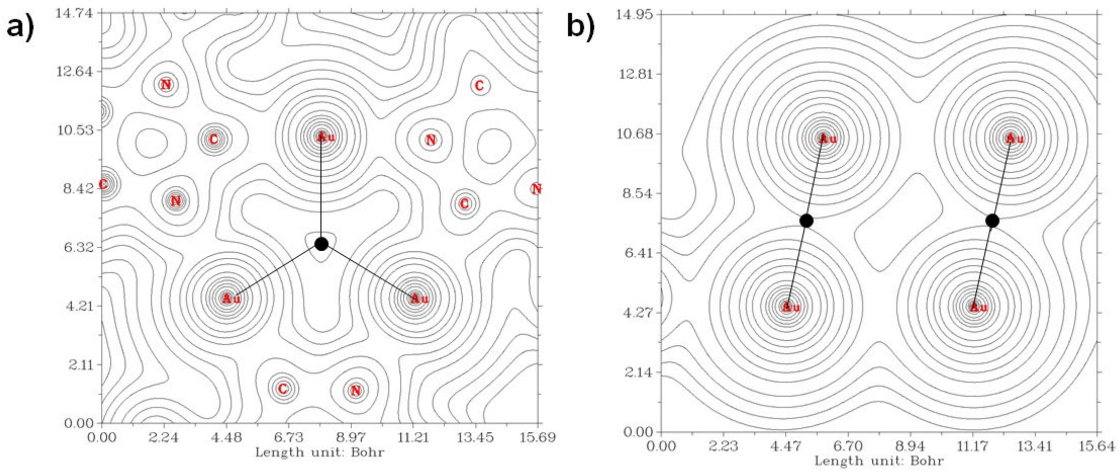

Figure S4. Electron density maps of $[Au_3(MeIm)]_3$-[1] through the planes containing the gold trimer (a), respectively dimer (b) and the corresponding bonding critical points. In particular (a) shows the missing BCS between gold atoms but the centered bonding in Au_3^{3+} triangles, whereas in intramolecular bonding between monomers in dimers, the BCS lays linear between gold atoms.

2. Hirshfeld surface analysis 2-D fingerprint plots of polymorph β

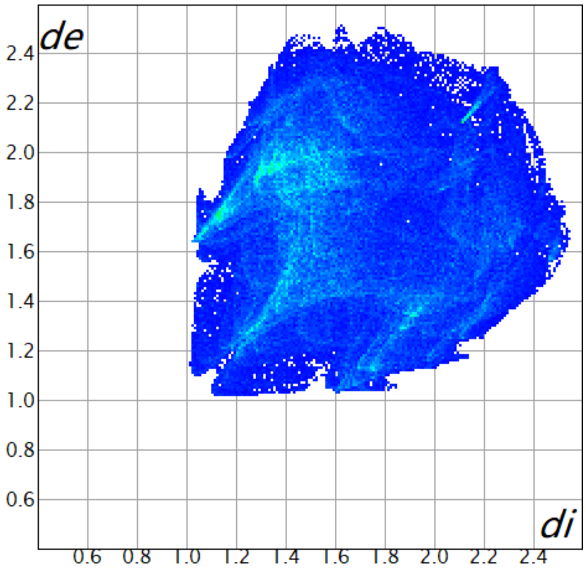

Figure S5. 2D fingerprint plot of dimer of trimer in Hirshfeld surface analysis.

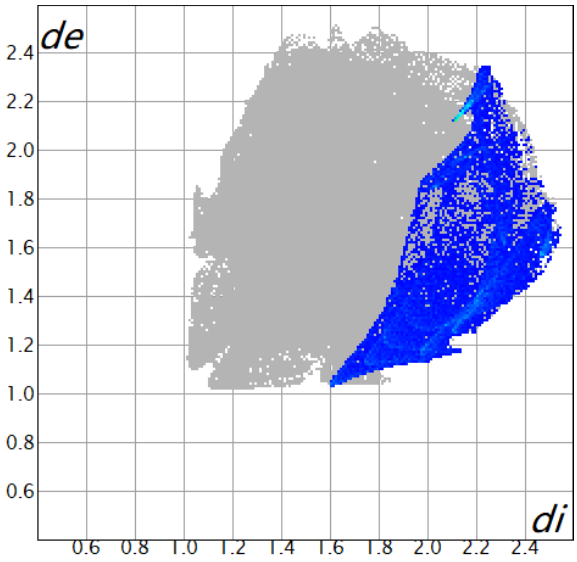

Figure S6. 2D fingerprint plot of dimer of trimer in Hirshfeld surface analysis. Close contacts of Au atoms to all outside atoms are highlighted, surface area included is 10.1%.

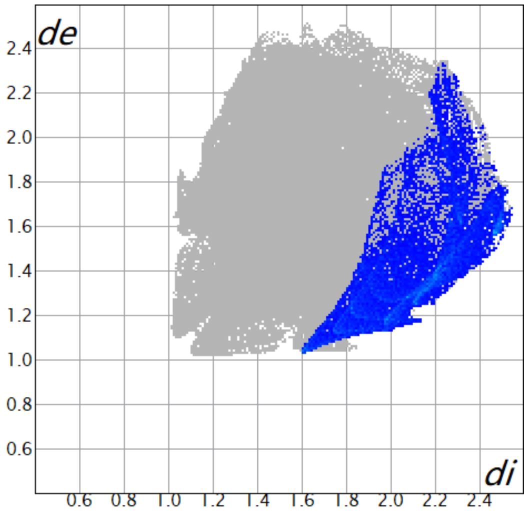

Figure S7. 2D fingerprint plot of dimer of trimer in Hirshfeld surface analysis. Close contacts of Au atoms to surrounding H atoms are highlighted, surface area included is 8.7%.

3. Crystallographic details

Data were collected on a Bruker D8 Venture single crystal x-ray diffractometer equipped with a CMOS detector (Bruker Photon-100), a TXS rotating anode (compound 21) with MoK$_\alpha$ radiation (λ = 0.71073 Å) and a Helios optic using the APEX3 software package.[7] Measurements were performed on single crystals coated with perfluorinated ether. The crystals were fixed on top of a kapton micro sampler and frozen under a stream of cold nitrogen. A matrix scan was used to determine the initial lattice parameters. Reflections were corrected for Lorentz and polarisation effects, scan speed, and background using SAINT. [8] Absorption correction, including odd and even ordered spherical harmonics was performed using SADABS.[8] Space group assignments were based upon systematic absences, E statistics, and successful refinement of the structures. The structures were solved using SHELXT with the aid of successive difference Fourier maps, and were refined against all data using SHELXL in conjunction with SHELXLE.[9-11] Hydrogen atoms were calculated in ideal positions as follows: Methyl H atoms were refined as part of rigid rotating groups, with a C–H distance of 0.98 Å and $U_{iso(H)} = 1.5 \cdot U_{eq(C)}$. Non-methyl H atoms were placed in calculated positions and refined using a riding model, with methylene, aromatic and other C–H distances of 0.99 Å, 0.95 Å and 1.00 Å, respectively, and $U_{iso(H)} = 1.2 \cdot U_{eq(C)}$. Non-hydrogen atoms were refined with anisotropic displacement parameters. Full-matrix least-squares refinements were carried out by minimizing $\Sigma w(F_o^2 - F_c^2)^2$ with the SHELXL weighting scheme.[10] Neutral atom scattering factors for all atoms and anomalous dispersion corrections for the non-hydrogen atoms were taken from *International Tables for Crystallography*.[12] Images of the crystal structures were generated with Mercury and PLATON.[13,14] CCDC XYZ contains the supplementary crystallographic data for this paper. These data are provided free of charge by The Cambridge Crystallographic Data Centre.

β-$Au_3(MeIm)_3$ (CCDC XYZ)

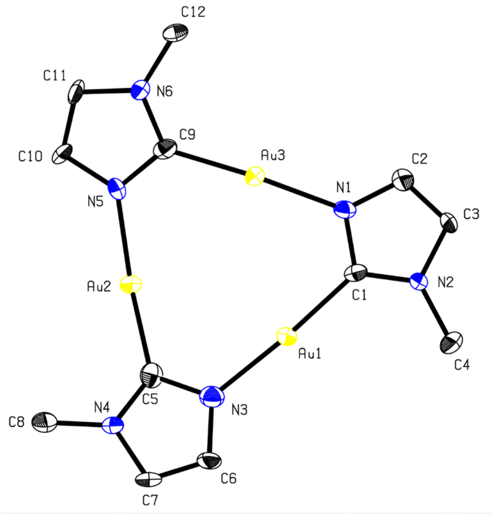

Diffractometer operator C. Jandl

scanspeed 2-20 s per frame

dx 40 mm

1918 frames measured in 10 data sets

phi-scans with delta_phi = 0.5

omega-scans with delta_omega = 0.5

shutterless mode

Crystal data

$C_{12}H_{15}Au_3N_6 \cdot CH_2Cl_2$	$F(000)$ = 816
M_r = 919.14	
Triclinic, $P\bar{1}$	D_x = 3.246 Mg m^{-3}
Hall symbol: -P 1	Melting point: ? K

a = 9.9981 (18) Å	Mo $K\alpha$ radiation, λ = 0.71073 Å
b = 10.0717 (17) Å	Cell parameters from 7084 reflections
c = 10.981 (2) Å	θ = 2.3–26.3°
α = 75.885 (5)°	μ = 23.63 mm^{-1}
β = 80.441 (6)°	T = 100 K
γ = 61.468 (5)°	Needle, colourless
V = 940.5 (3) $Å^3$	0.11 × 0.04 × 0.02 mm
Z = 2	

Data collection

Bruker Photon CMOS diffractometer	3709 independent reflections
Radiation source: TXS rotating anode	2880 reflections with $I > 2\sigma(I)$
Helios optic monochromator	R_{int} = 0.087
Detector resolution: 16 pixels mm^{-1}	θ_{max} = 26.0°, θ_{min} = 2.3°
phi– and ω–rotation scans	h = −12 12

Absorption correction: multi-scan
SADABS 2016/2, Bruker

$k = -12$ 12

T_{min} = 0.567, T_{max} = 0.745

$l = -13$ 13

21842 measured reflections

Refinement

Refinement on F^2

Secondary atom site location: difference Fourier map

Least-squares matrix: full

Hydrogen site location: inferred from neighbouring sites

$R[F^2 > 2\sigma(F^2)]$ = 0.034

H-atom parameters constrained

$wR(F^2)$ = 0.066

W = $1/[\Sigma^2(FO^2) + (0.0129P)^2 + 7.7003P]$ WHERE $P = (FO^2 + 2FC^2)/3$

S = 1.03

$(\Delta/\sigma)_{max}$ < 0.001

3709 reflections

$\Delta\rho_{max}$ = 1.83 e Å^{-3}

220 parameters

$\Delta\rho_{min}$ = −1.47 e Å^{-3}

0 restraints

Extinction correction: none

0 constraints

Extinction coefficient: -

Primary atom site location: iterative

α-$Au_3(MeIm)_3$ at 100K (CCDC XYZ), previously published by Ruiz *et al.* (CCDC 1955200)

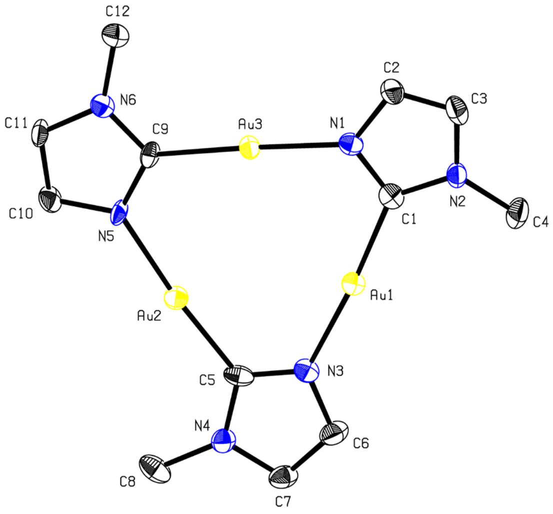

Diffractometer operator C. Jandl

scanspeed 1-3 s per frame

dx 80 mm

5670 frames measured in 18 data sets

phi-scans with delta_phi = 0.5

omega-scans with delta_omega = 0.5

shutterless mode

Crystal data

$2(C_{12}H_{15}Au_3N_6)$

M_r = 834.21	D_x = 3.547 Mg m^{-3}
Monoclinic, $P2_1/c$	Melting point: ? K
Hall symbol: -P 2ybc	Mo $K\alpha$ radiation, λ = 0.71073 Å

a = 19.925 (4) Å	Cell parameters from 9666 reflections
b = 7.8253 (17) Å	θ = 2.3–26.6°
c = 20.088 (4) Å	μ = 28.11 mm^{-1}
β = 94.030 (6)°	T = 100 K
V = 3124.4 (11) $Å^3$	Plate, colourless
Z = 8	0.28 × 0.07 × 0.02 mm
F(000) = 2928	

Data collection

Bruker D8 Venture diffractometer	6382 independent reflections
Radiation source: TXS rotating anode	5884 reflections with $I > 2\sigma(I)$
Helios optic monochromator	R_{int} = 0.068
Detector resolution: 16 pixels mm^{-1}	θ_{max} = 26.4°, θ_{min} = 2.3°
phi– and ω–rotation scans	h = −24 24
Absorption correction: multi-scan *SADABS* 2016/2, Bruker	k = −9 9

T_{min} = 0.433, T_{max} = 0.745

l = −25 25

115875 measured reflections

Refinement

Refinement on F^2

Secondary atom site location: difference Fourier map

Least-squares matrix: full

Hydrogen site location: inferred from neighbouring sites

$R[F^2 > 2\sigma(F^2)]$ = 0.027

H-atom parameters constrained

$wR(F^2)$ = 0.088

W = 1/[Σ²(*F*O²) + (0.0649*P*)² + 15.6336*P*] WHERE *P* = (*F*O² + 2*F*C²)/3

S = 1.03

$(\Delta/\sigma)_{max}$ = 0.002

6382 reflections

$\Delta\rho_{max}$ = 1.83 e Å^{-3}

385 parameters

$\Delta\rho_{min}$ = −1.98 e Å^{-3}

0 restraints

Extinction correction: none

0 constraints

Extinction coefficient: -

Primary atom site location: iterative

α-$Au_3(MeIm)_3$ at 295K (CCDC XYZ)

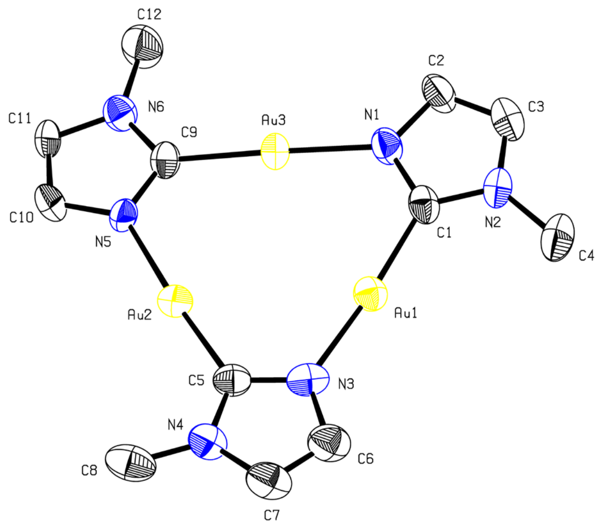

Diffractometer operator C. Jandl

scanspeed 1-5 s per frame

dx 40 mm

1290 frames measured in 6 data sets

phi-scans with delta_phi = 0.5

omega-scans with delta_omega = 0.5

shutterless mode

Crystal data

2($C_{12}H_{15}Au_3N_6$)

M_r = 834.21	D_x = 3.452 Mg m^{-3}
Monoclinic, $P2_1/c$	Melting point: ? K
Hall symbol: -P 2ybc	Mo $K\alpha$ radiation, λ = 0.71073 Å
a = 19.994 (5) Å	Cell parameters from 9446 reflections
b = 7.927 (2) Å	θ = 2.8–26.4°
c = 20.298 (5) Å	μ = 27.36 mm^{-1}
β = 93.814 (9)°	T = 293 K
V = 3210.0 (14) Å^3	Plate, colourless
Z = 8	0.28 × 0.08 × 0.03 mm
F(000) = 2928	

Data collection

Bruker D8 Venture diffractometer	6319 independent reflections

Radiation source: TXS rotating anode

5631 reflections with $I > 2\sigma(I)$

Helios optic monochromator

$R_{int} = 0.049$

Detector resolution: 16 pixels mm^{-1}

$\theta_{max} = 26.0°$, $\theta_{min} = 2.8°$

phi– and ω–rotation scans

$h = -24$ 24

Absorption correction: multi-scan *SADABS* 2016/2, Bruker

$k = -9$ 9

$T_{min} = 0.389$, $T_{max} = 0.745$

$l = -22$ 25

49266 measured reflections

Refinement

Refinement on F^2

Secondary atom site location: difference Fourier map

Least-squares matrix: full

Hydrogen site location: inferred from neighbouring sites

$R[F^2 > 2\sigma(F^2)] = 0.029$

H-atom parameters constrained

$wR(F^2) = 0.083$

$W = 1/[\Sigma^2(FO^2) + (0.0556P)^2 + 4.056P]$ WHERE $P = (FO^2 + 2FC^2)/3$

S = 1.07

$(\Delta/\sigma)_{max}$ = 0.002

6319 reflections

$\Delta\rho_{max}$ = 1.76 e Å^{-3}

386 parameters

$\Delta\rho_{min}$ = −1.83 e Å^{-3}

0 restraints

Extinction correction: *SHELXL2018*, $FC^{*}=KFC[1+0.001XFC^{2}\Lambda^{3}/SIN(2\Theta)]^{-1/4}$

0 constraints

Extinction coefficient: 0.00087 (5)

Primary atom site location: iterative

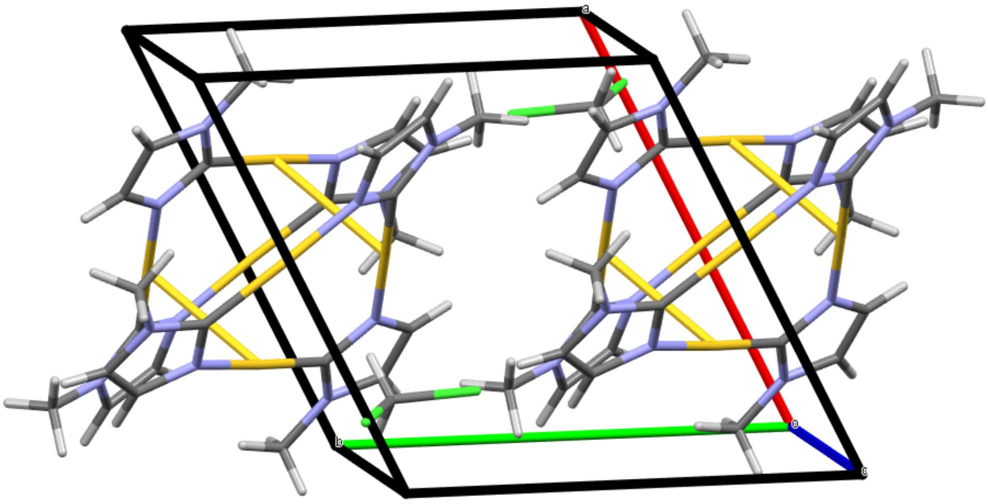

Figure S8. Packing diagram of β polymorph.

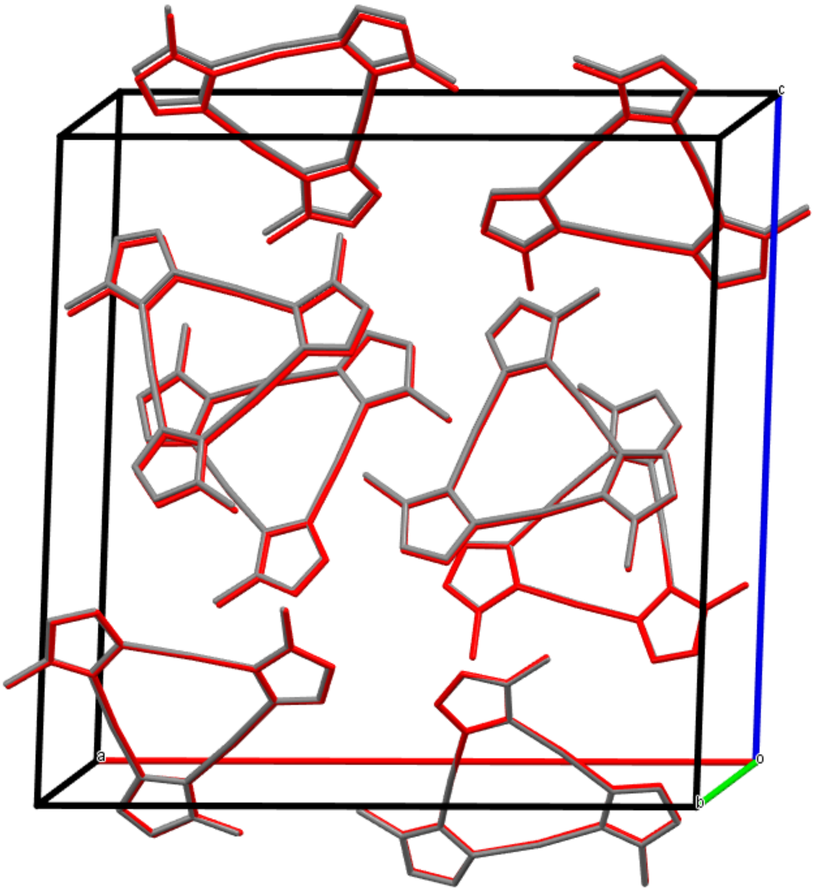

Figure S9. Overlay of the crystal structure of polymorph α (grey) and structure obtained by Ruiz *et.al.* (CCDC Deposition Number 1955200) (red), RMS=0.0396.

4. Spectral results

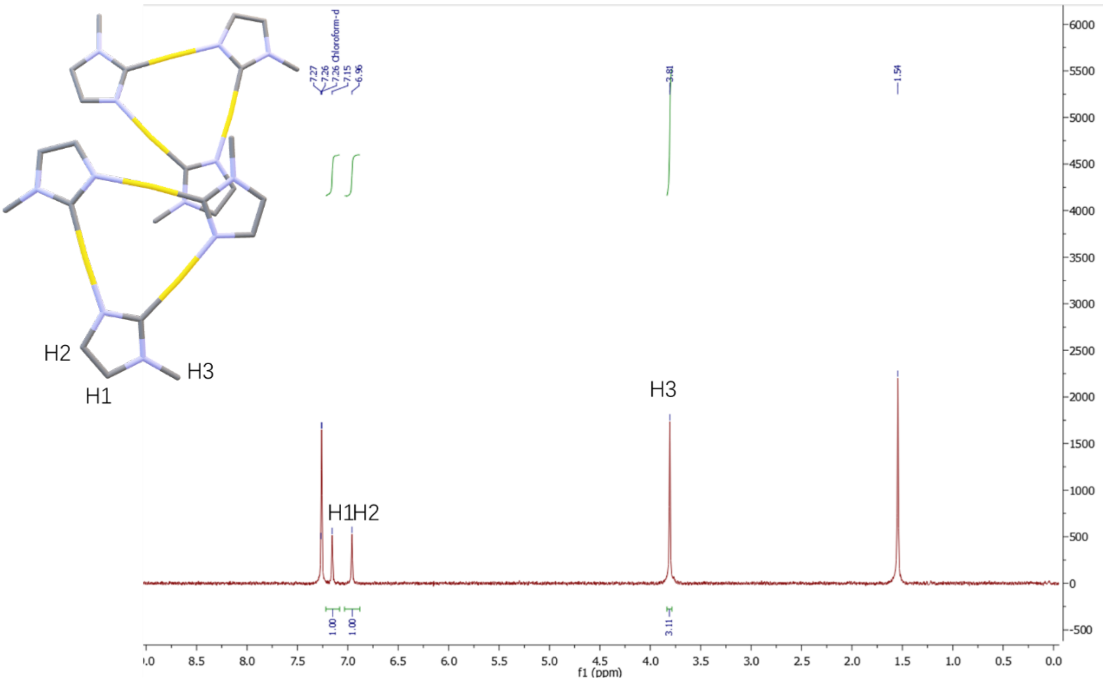

Figure S10. ^{1}H NMR spectrum (400 MHz, 298K) of polymorph α in CD_3Cl, peaks of solvent were marked, water peak can be found at 1.54 ppm.

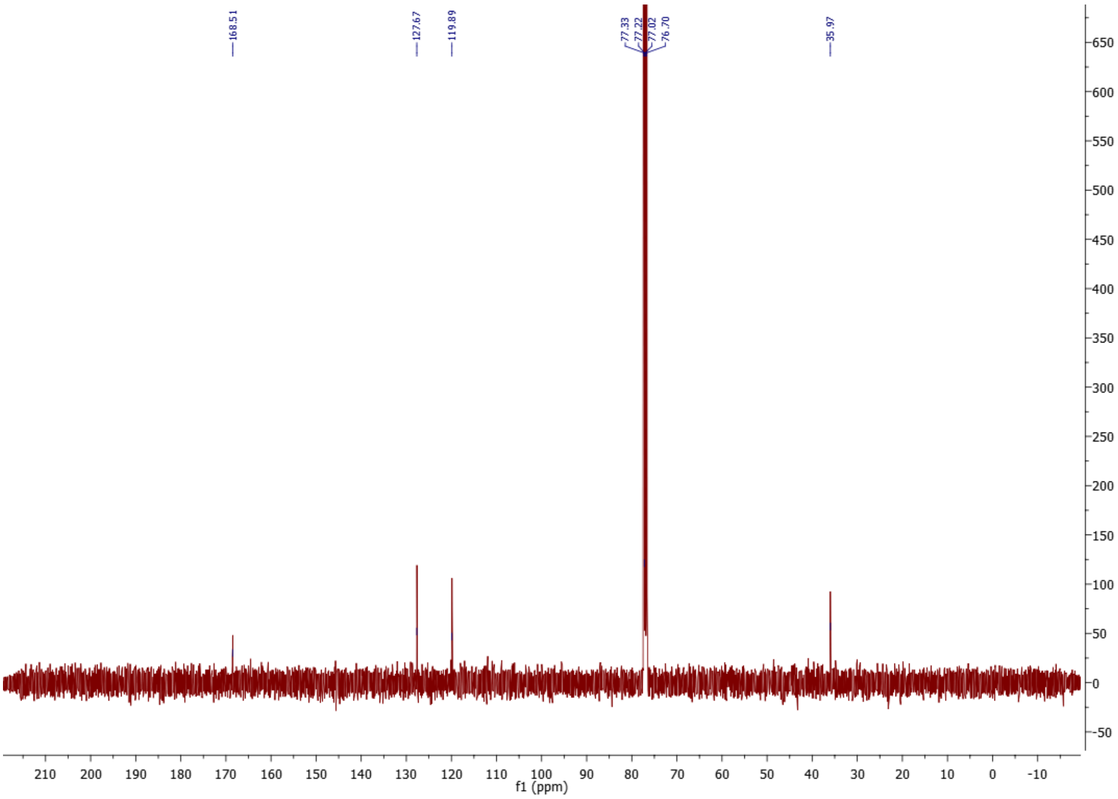

Figure S11. ^{13}C NMR spectrum (100 MHz, 298K) of polymorph α in CD_3Cl.

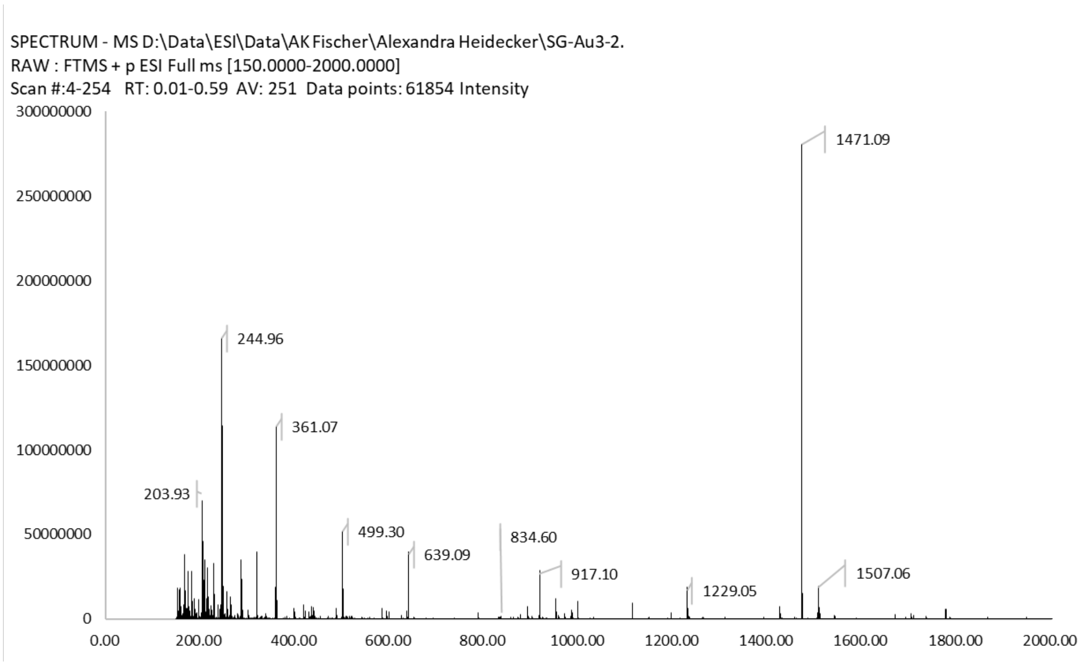

Figure S12. ESI-MS spectrum of $[Au_3(MeIm)]_3$.

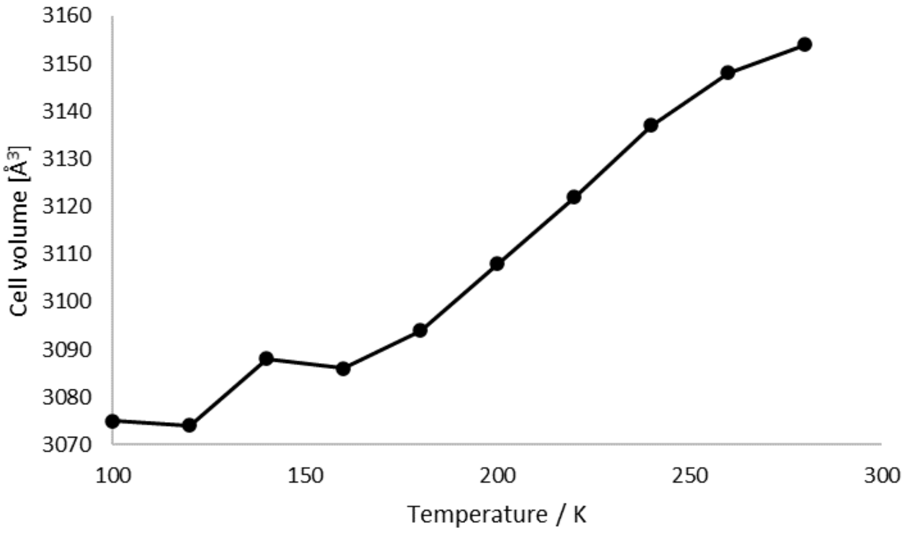

Figure S13. Measurement of unit cell volume versus temperature with single-crystal sample of polymorph $\boldsymbol{\alpha}$.

5. Solid-state emission spectra of polymorph α

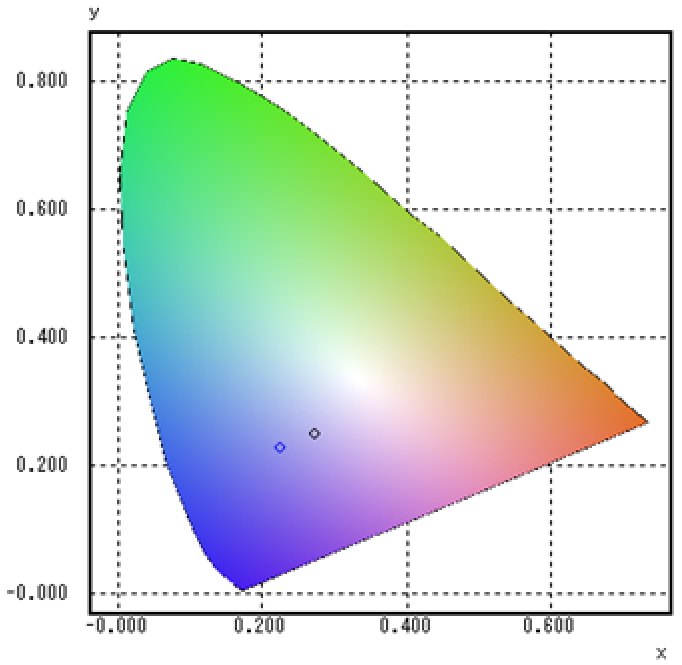

Figure S14. CIE colour space chromaticity diagram of polymorph **α** at room temperature with the emission circled in black, excitation circled in blue.

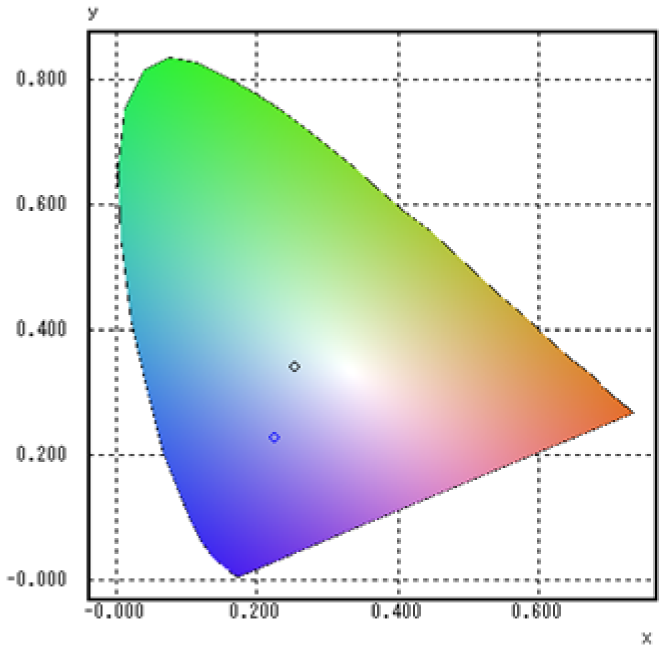

Figure S15. CIE colour space chromaticity diagram of polymorph α at cryogenic temperature with the emission circled in black, excitation circled in blue.

6. Reference

1. Frisch, M.J.; Trucks, G.W.; Schlegel, H.B.; Scuseria, G.E.; Robb, M.A.; Cheeseman, J.R.; Scalmani, G.; Barone, V.; Petersson, G.A.; Nakatsuji, H.; et al. *Gaussian 16 Rev. B.01*, Wallingford, CT, **2016**.
2. Zhao, Y.; Truhlar, D.G. The M06 suite of density functionals for main group thermochemistry, thermochemical kinetics, noncovalent interactions, excited states, and transition elements: two new functionals and systematic testing of four M06 functionals and 12 other functionals. *Theoretical Chemistry Accounts* **2008**, *119*, 525-525.
3. Stevens, W.J.; Basch, H.; Krauss, M. Compact effective potentials and efficient shared-exponent basis sets for the first- and second-row atoms. *The Journal of Chemical Physics* **1984**, *81*, 6026-6033.
4. Stevens, W.J.; Krauss, M.; Basch, H.; Jasien, P.G. Relativistic compact effective potentials and efficient, shared-exponent basis sets for the third-, fourth-, and fifth-row atoms. *Canadian Journal of Chemistry* **1992**, *70*, 612-630.
5. Cundari, T.R.; Stevens, W.J. Effective core potential methods for the lanthanides. *The Journal of Chemical Physics* **1993**, *98*, 5555-5565.
6. Lu, T.; Chen, F. Multiwfn: A multifunctional wavefunction analyzer. *Journal of Computational Chemistry* **2012**, *33*, 580-592.
7. Bruker AXS Inc., M., Wasconsin, USA. APEX suite of crystallographic software, APEX 3,Version 2015.5-2. **2015**.
8. Bruker AXS Inc., M., Wasconsin, USA. SAINT, Version 7.56 a and SADABS Version 2008/1. **2008**, *16*, 435-436.
9. Hübschle, C.B.; Sheldrick, G.M.; Dittrich, B. ShelXle: a Qt graphical user interface for SHELXL. *J Appl Crystallogr* **2011**, *44*, 1281-1284.
10. Sheldrick, G. *SHELXL-2014/7: program for the solution of crystal structures*, 2014.
11. Sheldrick, G. *SHELXL-97 (Release 97-2) University of Göttingen*, Germany: 1998.
12. Wilson, A.; Prince, E. Editor. International Tables for Crystallography, Vol. C. *Mathematical, Physical and Chemical Tables* **1992**.
13. Macrae, C.B.I.; Chisholm, J. A Edgington P. R McCabe P Pidcock E Rodriguez-Monge L Taylor R van de Streek J Wood P. *A J. Appl. Cryst* **2008**, *41*, 466.
14. Spek, A.L. Structure validation in chemical crystallography. *Acta Crystallogr Sect D Biol Crystallogr* **2009**, *65*, 148-155.
